# Exploring the Impact of Therapeutic Plasma Exchange on Organ Function in Patients With ACLF: A Retrospective, Single-center Propensity Score-matched Cohort Study

**DOI:** 10.1016/j.jceh.2025.102550

**Published:** 2025-03-24

**Authors:** Jonas Schumacher, Reinhard Henschler, Raymund Buhmann, Sirak Petros, Lorenz Weidhase, Rhea Veelken, Adam Herber, Janett Fischer, Thomas Berg

**Affiliations:** ∗ Division of Hepatology, Department of Medicine II, Leipzig University Medical Center, Leipzig, Germany; † Institute of Transfusion Medicine, University Hospital Leipzig, Leipzig, Germany; ‡ Medical ICU, University Hospital Leipzig, Leipzig, Germany

**Keywords:** acute on chronic liver failure, therapeutical plasma exchange, critical care, cirrhosis, liver support system

## Abstract

**Background and aims:**

Acute on chronic liver failure (ACLF) represents the most severe outcome of acute decompensation in patients with liver cirrhosis, with ACLF-grade 3 carrying a nearly 80% mortality rate within 28 days. Prognosis is especially dire within the first 3–7 days with full organ support. Currently, effective treatments are limited to transplantation, but therapeutic plasma exchange (TPE) may contribute by removing harmful inflammatory mediators and replenishing essential proteins, thus offering promise for patients unresponsive to standard medical treatments (SMT).

**Material and methods:**

This retrospective study analyzed patients with ACLF receiving SMT with or without TPE at a tertiary care transplant center. Patients were monitored for at least 90 days postdiagnosis. The primary endpoint was 28-day transplant-free survival, with secondary endpoints including 90-day transplant-free survival, organ dysfunction parameters, and chronic liver failure consortium (CLIF-C) scores. A cohort of 25 patients treated with SMT + TPE and 65 with SMT alone was identified. Propensity scores enabled 1:1 matching, resulting in a final analysis of 40 patients (20 TPE group and 20 SMT group).

**Results:**

Patients underwent a median of three (IQR 2.25–5) TPE sessions, starting a median of 14 (IQR 7.25–17) days after ACLF diagnosis. Significant improvements were observed in ACLF grade, CLIF-C-ACLF score, hepatic encephalopathy and prothrombin time 24–48 h postfinal TPE session. The 28-day transplant-free survival rates were 70% in the TPE group versus 45% in the SMT group (*P* = 0.083) and at 90 days, survival rates were 30% in bothgroups (*P* = 0.426). Patients unresponsive to SMT who received TPE had significantly higher 28-day transplant-free survival rates compared to those treated with SMT alone (70.6% vs 26.7%, *P* = 0.008).

**Conclusion:**

TPE may demonstrate potential efficacy in ameliorating organ dysfunction in patients with ACLF and could contribute to enhanced short-term survival in selected cases. However, clear criteria for initiating TPE have yet to be established. Unresponsiveness to standard medical treatment may serve as a potential surrogate parameter to guide clinical decision-making on an individual basis.

## Graphical abstract

**Figure:**
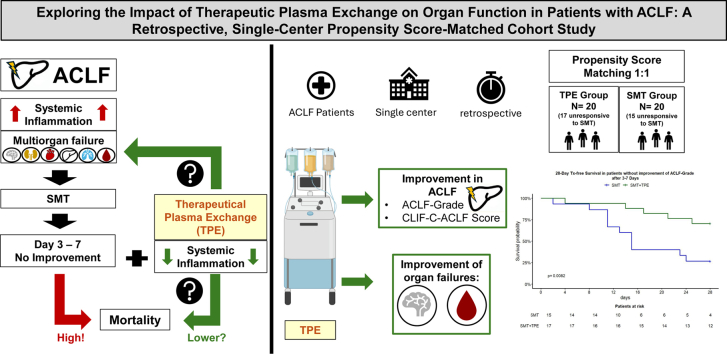

Acute on chronic liver failure (ACLF) represents the most severe sequelae of decompensated liver cirrhosis with short-term mortality ranging from 20% in ACLF grade 1 to approximately 80% in grade 3.^1^ A worsening of ACLF grade or chronic liver failure consortium criteria (CLIF-C)-ACLF scores after 3–7 days of full organ support is associated with even poorer outcomes.^2^ Thus, prognosis becomes critical after 3–7 days of standard medical treatment (SMT). Currently, liver transplantation (LT) is the only curative option, but it is often not feasible due to factors, such as uncontrolled infections, bleeding, or progression to ACLF grade 3b, which involves functional failure of more than three organ systems. These complications may necessitate delaying LT until organ functions stabilize to ensure an acceptable outcome.^3^ Therefore, therapies that enable a bridge to transplant or even a bridge to recovery are urgently needed.

The primary triggers of ACLF are infections and acute alcohol-related steatohepatitis,^4^^,^^5^ both of which are associated with severe inflammatory responses that are key drivers in the progression of ACLF.^6^^,^^7^ Accordingly, numerous inflammatory markers, pathogen- and damage-associated molecular patterns (PAMPS and DAMPS), have been associated with ACLF and mortality.^8^^,^^9^ Therapies that target systemic inflammation and neutralize harmful metabolites, are promising strategies in the management of ACLF.

Therapeutic plasma exchange (TPE) is a safe extracorporeal procedure that separates the patient's blood from its soluble components. Substitution is performed using fresh frozen plasma alone or in combination with albumin, with the ratio determined by global coagulation markers.^10^ TPE is an established treatment for diseases driven by antibodies and systemic inflammation. Beyond its well-known effects, TPE positively influences the immune system, coagulation, microcirculation, and endothelial permeability. These benefits might be partially attributed to the removal of proinflammatory cytokines, PAMPS and DAMPs, vascular endothelial growth factor, von Willebrand factor-multimers, and the replenishment of immunoglobulins, ADAMTS13, antithrombin III, and heparinase-2.^11^ In liver diseases, TPE has been shown to improve survival in acute liver failure and alleviate intractable hepatic pruritus.^10^ TPE also demonstrated a reduction in short-term mortality in sepsis.^12^ Although distinct, ACLF shares similar characteristics with sepsis, including multi-organ dysfunction and systemic inflammation, suggesting that TPE could offer similar benefits for patients with ACLF. Systematic reviews and expert opinions suggest favorable outcomes with TPE in acute liver failure and ACLF.^12^, ^13^, ^14^, ^15^, ^16^, ^17^, ^18^, ^19^ However, studies involving patients with ACLF are limited, often focused on hepatitis B-related ACLF, and typically use the Asian Pacific Association for Study of the Liver (APASL) criteria to define ACLF.^20^, ^21^, ^22^, ^23^, ^24^, ^25^ Evidence for TPE in ACLF defined by the European Association for the Study of the Liver (EASL)-CLIF criteria in Western populations is scarce, and patient selection criteria for TPE initiation remain undefined.

In this study we aimed to evaluate the effects of TPE in ACLF and assess transplant-free survival rates in those unresponsive to SMT. The primary endpoint was 28-day transplant-free survival, while secondary endpoints included 90-day transplant-free survival, changes in organ function parameters, and CLIF-C scores in patients treated with TPE.

## Patients and methods

### Patient Inclusion Criteria

**Figure 1:**
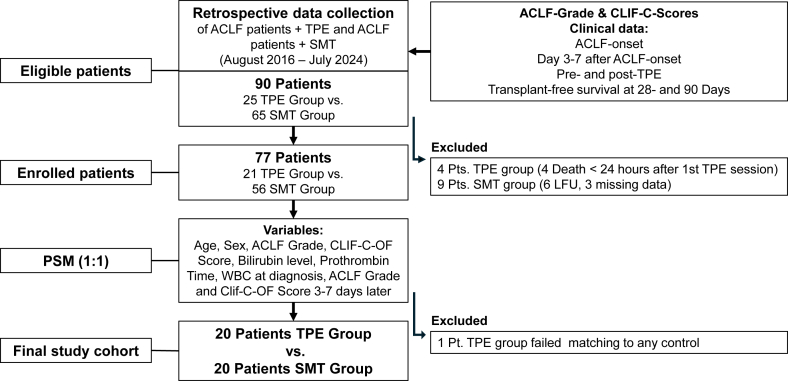
Study-flow chart. ACLF; acute on chronic liver failure; CLIF-C, chronic liver failure consortium; LFU, lost to follow-up; OF, organ failure; Pt, patient; SMT, therapeutical plasma exchange; TPE, therapeutical plasma exchange; WBC, white blood cell count

In this retrospective cohort study at a European tertiary care transplant center, we included all patients over 18 with ACLF, as defined by the European Foundation for the Chronic Liver Failure consortium (EF-CLIF-C) criteria who received TPE treatment between August 2016 and July 2024 (TPE group). To be included in the study, patients had to survive at least 24 h after the first session of TPE. Patients with ACLF who did not receive TPE treatment served as controls (SMT group). Exclusion criteria included participation in an interventional clinical trial, active extrahepatic malignancy, or hepatocellular carcinoma beyond Milan criteria. Clinical data were collected by chart review. Patients were monitored for at least 90 days after the onset of ACLF. A total of 25 patients were identified in the TPE group and 65 in the SMT group. Four patients in the TPE group were excluded due to death within 24 h after TPE initiation from uncontrolled septic shock. In the SMT group, six patients were lost to follow-up within 90 days of ACLF onset, and three were excluded due to incomplete laboratory and clinical data, which prevented EF-CLIF score calculation. Propensity score matching (PSM) was used to match the remaining 21 patients in the TPE group to the SMT group in a 1:1 ratio (Figure 1). In our study, we utilized PSM to pair patients with controls based on similarity in probability scores calculated from a predefined set of variables. A critical step was setting the caliper at 0.2. This match tolerance, or the maximum allowable difference in propensity scores between matched individuals, was specifically chosen to ensure precise matching, while maintaining an adequate number of matched pairs. This approach is known to minimize bias from uncontrolled confounding factors and supports more reliable causal inference.^26^

PSM with the match tolerance of 0.2 (26) was adjusted for age, sex, ACLF grade, CLIF-C organ failure (OF) score, white blood cell count (WBC), bilirubin, and prothrombin time (PT) serum levels at diagnosis and ACLF grade and CLIF-C-OF score 3–7 days after the onset of ACLF. One patient in the TPE group could not be matched to any control, resulting in 40 patients for the final statistical analysis. Worsening or lack of improvement of ACLF grade after a maximum of 7 days of SMT was classified as unresponsive to SMT. In both groups patients received at least 3–7 days of SMT prior to TPE initiation, death, or LT, respectively.

The study was conducted in accordance with the Declaration of Helsinki and its subsequent amendments.

### Treatment Procedures

#### Standard Medical Treatment

SMT for ACLF involved identifying potential precipitants at presentation, administering antimicrobial treatments for suspected and confirmed infections, and using corticosteroids in cases of acute alcohol-related steatohepatitis. Albumin substitution was performed in cases of spontaneous bacterial peritonitis, hepatorenal syndrome, and during large volume paracentesis. Additionally, SMT encompassed renal replacement therapy, mechanical ventilation, and the use of inotropes. Overall, the management of ACLF followed the German and European guidelines for handling ACLF and decompensated cirrhosis.^27^, ^28^, ^29^

#### Therapeutic Plasma Exchange

After obtaining informed consent, TPE was conducted using a continuous-flow centrifugation system, specifically the Spectra Optia Apheresis System (Terumo BCT, Inc.). For anticoagulation, a 12:1 ratio of acid citrate dextrose formula A was used. To prevent citrate toxicity, calcium gluconate was administered intravenously, and calcium levels were routinely monitored. The patient's total blood volume was determined using the Nadler formula, as referenced in the citation.^30^ Each session involved the replacement of 1–1.5 times the calculated plasma volume with replacement fluids comprising 5% albumin and/or fresh frozen plasma. Preference was given to plasma due to the prevalent coagulopathy in these patients. Substitution fluid management during TPE adhered to local standard operating procedures. Specifically, a 1:1 mixture of plasma and 5% albumin was used in patients with cirrhosis when fibrinogen serum levels ranged between 1.5 and 2 g/l, provided there was no bleeding in the previous 24 h. In cases where bleeding occurred within the last 24 h or fibrinogen serum levels were below 1.5 g/l, 100% plasma was utilized. TPE was initially planned to be conducted daily; however, due to organizational constraints, some patients received treatment every other day. All TPE procedures were carried out by operators trained specifically in TPE, under close medical supervision. Detailed procedural information can be found in Table S1. The decision to initiate TPE was made by the treating medical team on a case-by-case basis, reflecting a tailored approach rather than a standardized protocol (Table 2).

### Statistics

Differences between the TPE group and SMT group in CLIF-C-scoring systems, organ function parameters, and laboratory values at baseline and at a maximum of 7 days after the onset of ACLF were compared using Mann–Whitney U test and Fisher exact test if applicable. Survival was estimated using Kaplan–Meier curves. Survival functions were calculated via log-rank-test. Pre-TPE data were collected either on the day before or on the day of TPE-initiation. Post-TPE data were collected 24–48 h and again 5–7 days after the final TPE session. Wilcoxon test was used for metric and ordinal-scaled variables. McNemar test was applied to dichotomic variables. Hedge's g with bias correction was used to calculate standardized mean differences. Results were considered significant if a two-sided alpha <0.05 was reached.

Statistical tests were performed using SPSS-29 and R (R Core Team, 2024). GraphPad Prism 8.0 (GraphPad Software, San Diego, CA, USA) and R (R Core Team, 2024) were used for the preparation of the figures.

## Results

### Baseline Characteristics and Outcomes Before and After Propensity Score Matching

Before PSM, the TPE group and SMT group demonstrated statistically significant differences regarding baseline white blood cell count, ACLF grade and CLIF-C-OF score at 3–7 days of SMT (Table 1). After PSM, there were no statistically significant differences between both groups regarding age, sex, CLIF-C scoring systems, serum levels of PT, bilirubin, creatinine, WBC, presence and graduation of hepatic encephalopathy (HE), need for renal replacement therapy (RRT), vasopressors, mechanical ventilation at baseline, and CLIF-C scores after 3–7 days of SMT (Table 2). The most common etiology of cirrhosis was alcohol-related liver disease in 17 out of 20 patients in the TPE group and 14 out of 20 patients in the SMT group. The median age of patients in the TPE group was 51 (36.5–64.25) years vs 53.5 (35.75–59.75) years in the SMT group.

**Table 1:** Patient Characteristics of the Total Patient Cohort and Prematching.

Parameter (unit)	N	Group 1: SMT + TPE, n = 21 (Median + IQR or %/n)	Group 2: SMT, n = 56 (Median + IQR or %/n)	*P*-value	SMD (CI 95%)
Age (years)	77	49 (38–63.5)	57 (47–63)	0.252	0.35 (−0.17-0.86
Sex	77	61.9% male	73.21% male	0.405	–
ACLF grade baseline	77	2 (1–3)	2 (1–2)	0.408	−0.21 (−0.71-0.29)
CLIF-C-ACLF score baseline	76	48 (44–55)	49 (43–54)	0.940	−0,02 (−0.52-0.49)
CLIF-C-OF score baseline	77	10 (9–11)	10 (8.25–11)	0.753	0.12 (−0.63-0.37)
WBC baseline (exp9/l)	77	13.9 (10.45–19.8)	10.75 (6.93–15.88)	0.042	−0.39 (−0.90-0.11)
Bilirubin level baseline (μmol/l)	77	363 (115.4–473.15)	162.75 (40.1–320.05)	0.059	−0.49 (−1.01-0.02)
Prothrombin time baseline (%)	76	38 (30–51.5)	32 (25–53)	0.353	−0.13 (−0.61-0.35)
Creatinine baseline (μmol/l)	77	207 (147–302.5	188.5 (130–233.5)	0.272	−0.32 (−0.85-0.22)
HE baseline (West Haven)	77	0 (0–1.5)	0 (0–2)	0.662	0.08 (−0.41-0.47)
RRT baseline	77	0% (0/20)	3.57% (2/56)	1	–
Need for vasopressors baseline	77	14.3% (3/20)	19.6% (11/56)	0.562	–
Mechanical ventilation baseline	77	4.8% (1/20)	16.07% (9/56)	0.270	–
ACLF grade day 3–7	77	2 (1–3)	2 (1–2)	0.049	−0.5 (−0.99-0)
CLIF-C-ACLF score day 3–7	76	54 (45–60)	51 (35–63)	0.192	−0.36 (−0.84-0.12)
CLIF-C-OF score D 3-7	77	11 (10–13.5)	10 (8–11)	0.011	−0.63 (−1.16-−0.1)

ACLF, acute on chronic liver failure; CI, confidence interval; CLIF-C, chronic liver failure consortium; HE, hepatic encephalopathy; MASLD, metabolic dysfunction-associated steatotic liver disease; OF, organ failure; PT, prothrombin time; RRT, renal replacement therapy; SMD, standardized mean deviation TPE, therapeutical plasma exchange; WBC, white blood cell.

Patients in the TPE group received a median of 3 (IQR 2.25–5) TPE sessions, initiated a median of 14 (IQR 7.25–17) days after ACLF diagnosis. One patient in the TPE group and five patients in the SMT group underwent LT. By the study's end, six patients in each group were still alive. The most common causes of death were sepsis and progression of ACLF with multiorgan dysfunction (55% in each group). Each group had one fatal hemorrhage associated with a procedure: one occurred after pleural drainage in the TPE group, and the other followed hernia surgery in the SMT group. No bleeding events were associated with TPE. Detailed patient characteristics are provided in Table 1, Table 2.

**Table 2:** Patients Characteristics After Propensity Score Matching.

Parameter (unit)	Group 1: SMT + TPE, n = 20 (Median IQR]	Group 2: SMT, n = 20 (Median IQR)	*P*-value	SMD (CI 95%)
Age (years)	51 (36.5–64.25)	53.5 (35.75–59.75)	0.871	0.01 (−0.59-0.62)
Sex	60% male	60% male	1	–
ACLF-grade	2 (1–2.75)	2 (2–3)	0.385	0.27 (−0.33-0.88)
CLIF-C-ACLF score	48 (44–53.75)	52.5 (45–54.75)	0.350	0.2 (−0.41-0.81)
CLIF-C-OF score	10 (9–11)	10 (9.25–11.75)	0.437	0.2 (−0.41-0.81)
WBC (exp9/l)	13.8 (10.38–18.58)	11.55 (9.85–18.78)	0.583	−0.12 (−0.73-0.48)
HE (West Haven)	0 (0–1)	0.5 (0–2)	0.327	0.31 (−0.31-0.92)
PT (%)	38.5 (30–53.25)	31.5 (23.5–37.5)	0.140	−0.34 (−0.95-0.28)
Bilirubin (μmol/l)	315 (112.7–456.5)	246.4 (110.2–490.7)	0.738	−0.06 (−0.54-0.67)
RRT (% of cohort)	0% (0/20)	5% (1/20	1	–
Vasopressors (% of cohort)	15% (3/20)	25% (5/20)	0.449	–
Mechanical ventilation (% of cohort)	5% (1/20)	15% (3/20)	0.605	–
ACLF-grade day 3–7	2 (1–3)	2 (1–3)	0.776	−0.09 (−0.7-0.51)
CLIF-C-ACLF-score day 3–7	54 (44.5–59.25)	53 (46–61.25)	0.978	0.04 (−0.55-0.66
CLIF-C-OF score day 3–7	11 (10–12.75)	10 (9.25–13.25)	0.419	−0.13 (−0.74-0.48)
Etiology of cirrhosis (n)			–	–
- Alcohol-related liver disease	14/20	17/20		
- MASLD	1/20	0		
- AILD	1/20 (AIH)	1/20 (PBC)		
- Unknown	4/20	2/20		
ACLF - precipitant	- Unknown 7/20 - Alcohol-related steatohepatitis 6/20 - SBP 2/20 - Variceal bleeding 1/20 - Duodenal ulcer 1/20 - Post-surgery ○ Hip fracture 1/20 ○ Partial gastrectomy for gastric ulcera 1/20 ○ Hemihepatectomy for HCC 1/20	- Unknown 7/20 - Alcohol-related steatohepatitis 2/20 - SBSI 1/20 - Pneumonia 6/20 - SBP 2/20 - Soft-tissue infection 1/20 - Post-interventional ○ cardiac catheterization 1/20	–	–
**Plasma exchange modalities**				
Days from ACLF diagnosis to TPE initiation	14 (IQR 7.25–17)	–	–	–
Number of TPE-sessions	3 (IQR 2.25–5)	–	–	–
Reason to initiale TPE	- Treatment for alcohol-related steatohepatitis 4/20 - Severe HE without response to conservative treatment 2/20 - Unknown 3/20 - Suspected TMA 4/20 - AILD unresponsive to medical treatment 1/20 - Bridge to recovery concept 6/20	–	–	–
**Clinical outcomes**				
Liver transplantation (n)	1/20	5/20	0.18	–
Death at end of omission (n)	14/20	14/20	1	–
Alive >90 days post-ACLF diagnosis (n)	6/20	6/20	1	–
Cause of death (n)1/20			–	–
- Sepsis	5/20	7/20		
- Disease progression	6/20	4/20		
- De-escalation of therapy according to the patients will	2/20	2/20		
- Hemorrhage after surgery/intervention	1/20	1/20		

ACLF, acute on chronic liver failure; AILD, autoimmune liver disease; CI, confidence interval; CLIF-C, chronic liver failure consortium; HE, hepatic encephalopathy; MASLD, metabolic dysfunction-associated steatotic liver disease; OF, organ failure; PT, prothrombin time; RRT, renal replacement therapy; SBP, spontaneous bacterial peritonitis; SBSI, spontaneous blood stream infection; SMD, standardized mean deviation; SMT, standard medical treatment; TMA, thrombotic microangiopathy; TPE, therapeutical plasma exchange; WBC, white blood cell.

### Survival Analysis and Treatment Response Evaluation

**Figure 2:**
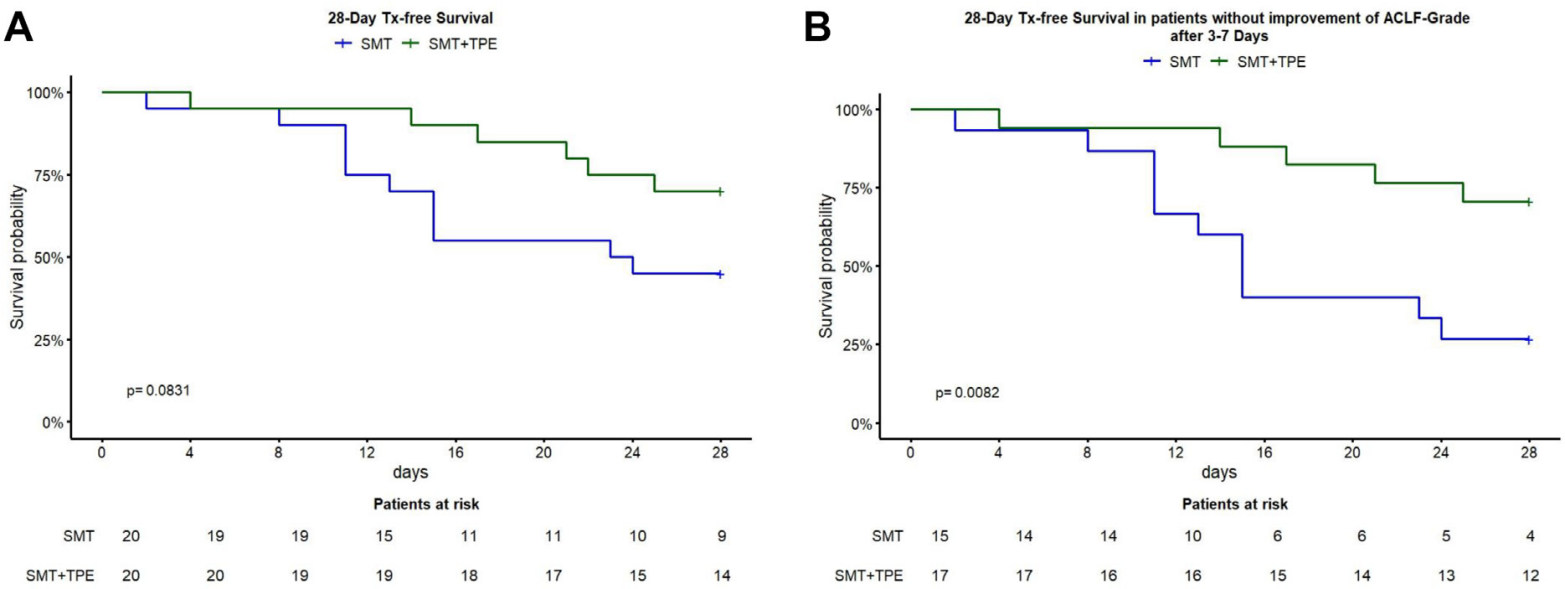
**A**: 28-day transplant-free survival rates of the whole study cohort irrespective of response to SMT after 3–7 days. SMT, standard medical treatment. **B**: 28-day transplant-free survival rates were analyzed for patients unresponsive to standard medical treatment (SMT) after 3–7 days of SMT in both the therapeutic plasma exchange (TPE) group—prior to any TPE application—and the SMT group.

**Figure 3:**
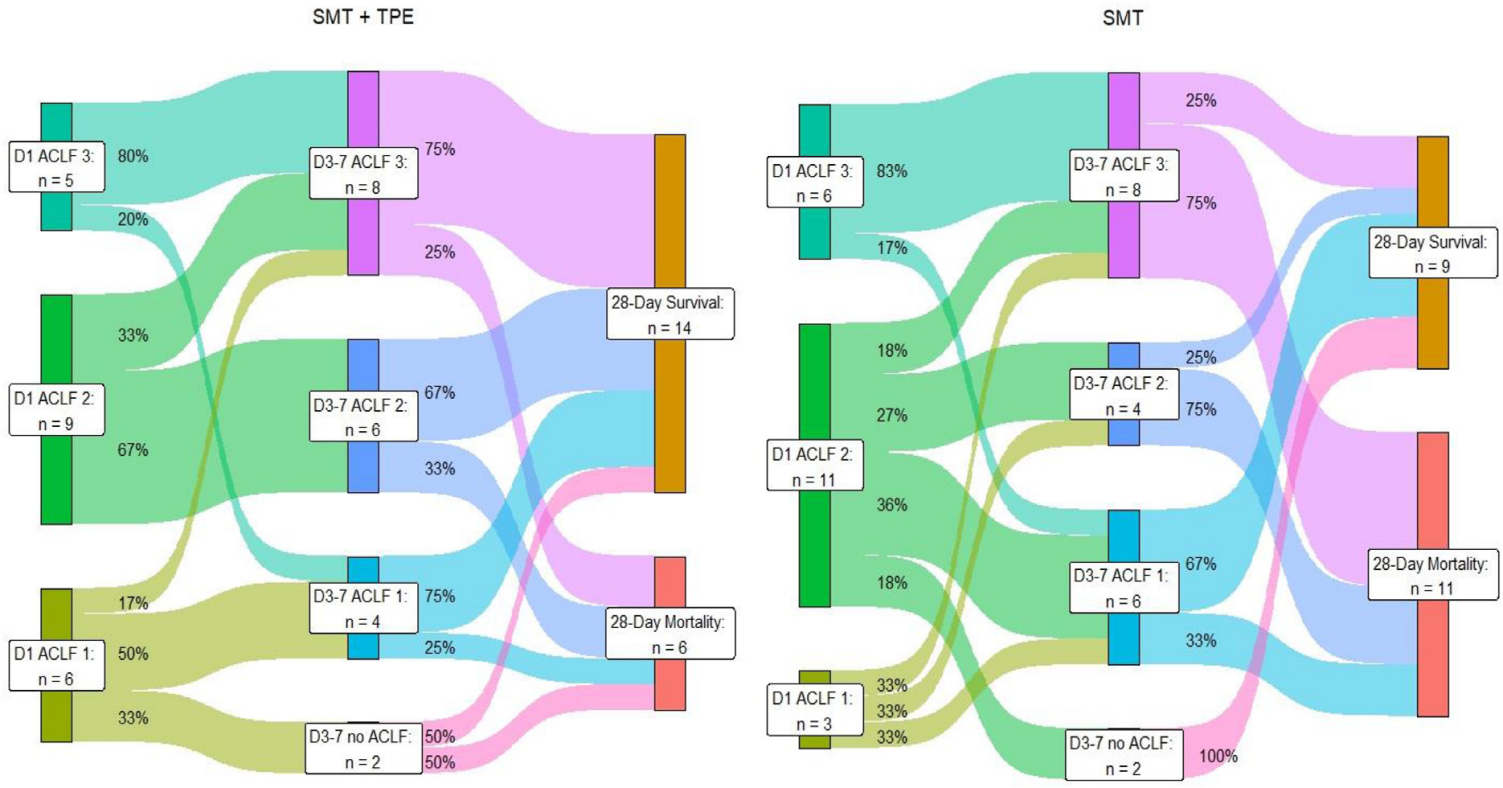
ACLF trajectories: The sankey plot show the distribution of patients with ACLF grade 1–3 at baseline, after 3–7 days of SMT, and the transplant-free survival rates after 28 days for the SMT + TPE group (left) and SMT group (right). ACLF, acute on chronic liver failure; SMT, standard medical treatment; TPE, therapeutic plasma exchange.

The 28-day transplant-free survival rates were 70% (14/20) in the TPE group versus 45% (9/20) in the SMT group (*P* = 0.083 log rank test) and at 90 days, survival rates were 30% (6/20) in both groups (*P* = 0.426 log rank test) (Figure 2A and Fig. S2A). Seventeen patients in the TPE group and 15 patients in the SMT group were unresponsive to SMT. In both groups, two patients recovered from ACLF by the time of reassessment of ACLF grade (3–7 days after ACLF diagnosis). The trajectory pathways of ACLF in both groups regarding 28-day transplant-free survival are shown in Figure 3 and regarding 90-day transplant-free survival in Fig. S1.

Patients in the TPE group who did not show improvement in ACLF after a maximum of 7 days of SMT prior to initiation of TPE had significantly improved 28-day transplant-free survival rates compared to patients in the SMT group who did not show improvement in ACLF grade after a maximum of 7 days of SMT (Figure 2B). Univariate Cox regression analysis showed that HE and the need for vasopressors at baseline increased the risk of death, whereas TPE was associated with a risk reduction (HR 0.256, *P* = 0.015) at 28 days. However, when patients were followed for an extended period of 90 days, TPE demonstrated no effect on 90-day mortality (HR 0.474, *P* = 0.078) (Table 3A, Table 3BA and B). Multivariate analysis, which included TPE treatment and baseline CLIF-C scores as well as scores after a maximum of 7 days of SMT, showed a reduction in the risk of death within the TPE cohort at 28 days (HR 0.313, *P* = 0.042). However, this risk reduction was not observed at 90 days (HR 0.518, *P* = 0.149), as detailed in Table 4A, Table 4BA and B.

**Table 3A:** Results of Univariate Cox Regression Analysis in the SMT Group and TPE Group Regarding 28-Day Mortality in the Subgroup of Patients Without Response to Standard Medical Treatment.

Variable	HR (CI 95%) 28-day mortality	*P*-value
TPE	0.256 (0.091–0.773)	0.015
Age	1.004 (0.968–1.042	0.817
Sex	0.861 (0.32–2.314)	0.766
ACLF-grade	1.488 (0.719–3.079	0.284
CLIF-C-ACLF score	1.061 (0.997–1.129)	0.063
CLIF-C-OF score	1.293 (0.987–1.694	0.062
HE	1.593 (1.051–2.416	0.028
WBC	0.974 (0.914–1.038	0.419
Bilirubin	0.999 (0.996–1.001)	0.293
PT	0.989 (0.957–1.022)	0.518
Renal replacement therapy	5.792 (0.677–45.592)	0.109
Vasopressor support	2.054 (1.14–3.703	0.017
Mechanical ventilation	3.494 (0.959–12,723)	0.058
ACLF grade day 7	1.017 (0.525–1.971	0.960
CLIF-C-ACLF score day 7	1.048 (0.998–1.1	0.06
CLIF-C-OF score day 7	1.163 (0.936–1.445)	0.174

ACLF, acute on chronic liver failure; CI, confidence interval; CLIF-C, chronic liver failure consortium; HE, hepatic encephalopathy; HR, hazard ratio; OF, organ failure; PT, prothrombin time; SMT, standard medical treatment; TPE, therapeutical plasma exchange; WBC, white blood cell.

**Table 3B:** Results of Univariate Cox Regression Analysis in the SMT Group and TPE Group Regarding 90-Day Mortality in the Subgroup of Patients Without Response to Standard Medical Treatment.

Variable	HR (CI 95%) 90-day mortality	*P*-value
TPE	0.474 (0.207–1.087)	0.078
Age	1.009 (0.978–1.041)	0.566
Sex	0.644 (0.278–1.494	0.306
ACLF grade	1.278 (0.702–2.328)	0.422
CLIF-C-ACLF score	1.06 (1.001–1.121)	0.045
CLIF-C-OF score	1.248 (0.982–1.586	0.07
HE	1.312 (0.876–1.965	0.187
WBC	0.97 (0.923–1.02)	0.235
Bilirubin	0.999 (0.997–1.001)	0.283
Prothrombin time	0.994 (0.968–1.020)	0.656
Renal replacement therapy	5.792 (0.677–45.592)	0.109
Vasopressor support	2.117 (1.263–3.549)	0.004
Mechanical ventilation	3.494 (0.959–12.723)	0.058
ACLF grade day 7	1.085 (0.627–1.880)	0.770
CLIF-C-ACLF score day 7	1.059 (1.012–1.108	0.012
CLIF-C–OF–score day 7	1.201 (0.995–1.449)	0.057

ACLF, acute on chronic liver failure; CI, confidence interval; CLIF-C, chronic liver failure consortium; HE, hepatic encephalopathy; HR, hazard ratio; OF, organ failure; PT, prothrombin time; TPE, therapeutical plasma exchange; WBC, white blood cell.

**Table 4A:** Results of Multivariate Cox Regression Analysis in the SMT Group and TPE Group Regarding 28-Day Mortality in the Subgroup of Patients Without Response to Standard Medical Treatment.

Variable	HR (CI 95%) 28-Day Mortality	*P*-value
TPE	0.313 (0.102–0.957)	0.042
CLIF-C-ACLF score	0.946 (0.818–1.095)	0.458
CLIF-C-OF score	1.417 (0.803–2.503)	0.229
CLIF-C-ACLF score day 7	1.104 (0.960–1.270)	0.164
CLIF-C–OF–score day 7	0.753 (0.410–1.382)	0.360

ACLF, acute on chronic liver failure; CI, confidence interval; CLIF-C, chronic liver failure consortium; HR, hazard ratio; OF, organ failure; TPE, therapeutical plasma exchange.

**Table 4B:** Results of Multivariate Cox Regression Analysis in the SMT Group and TPE Group Regarding 90-Day Mortality in the Subgroup of Patients Without Response to Standard Medical Treatment.

Variable	HR (CI 95%) 90-Day Mortality	*P*-value
TPE	0.518 (0.212–1.266)	0.149
CLIF-C-ACLF score	0.950 (0.836–1.080)	0.433
CLIF-C-OF score	1.278 (0.765–2.138)	0.349
CLIF-C-ACLF score day 7	1.099 (0.972–1.243)	0.131
CLIF-C–OF–score day 7	0.846 (0.5–1.432)	0.522

ACLF, acute on chronic liver failure; CI, confidence interval; CLIF-C, chronic liver failure consortium; HR, hazard ratio; OF, organ failure; TPE, therapeutical plasma exchange.

### Post-TPE Reassessment of Clinical and Laboratory Parameters

**Figure 4:**
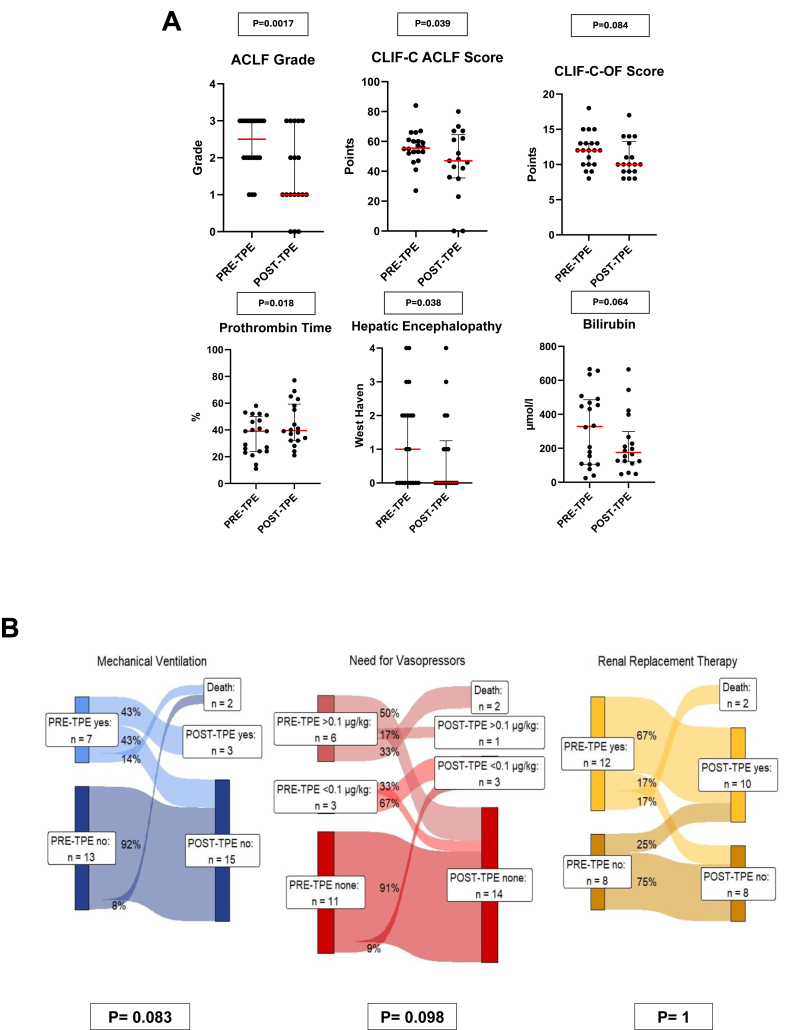
Changes in organ dysfunction parameters and need for organ support in the TPE group before TPE and 24–48 h after the last TPE application. **A.** The scatter plots in the upper two rows show the median changes (red bars) and individual values (black dots) of the pre- and post-TPE CLIF-C scores and organ function parameters (TPE group). **B.** The sankey plots in the lower row show the proportion of patients in the TPE group needing (from left to right) mechanical ventilation (blue), vasopressors (red), and RRT (yellow) pre- and post-TPE. The need for vasopressors is subdivided into the proportion of patients with no need for vasopressors (none), low dose (<0.1 μg/kg/min), and high dose (>0.1 μg/kg/min). TPE, therapeutical plasma exchange; CLIF-C, chronic liver failure consortium; RRT, renal replacement therapy.

Reassessment of CLIF-C-scoring parameters and laboratory values 24–48 h after the final TPE session was available in 17 out of 20 patients in the TPE group. This evaluation included the following parameters: ACLF grade, CLIF-C-ACLF score, HE, PT, CLIF-C-OF score, serum bilirubin levels, and the need for vasopressors, mechanical ventilation, and RRT. Improvements were observed in all listed parameters after TPE, with statistically significant changes in ACLF grade, CLIF-C-ACLF score, HE, and PT. Trends toward improvement were also noted in other parameters, though not reaching statistical significance (Table 5). The detailed results are presented in Figure 4A and 4B. A second reassessment of CLIF-C-scoring parameters and laboratory values, conducted 5–7 days after the final TPE session, revealed statistically significant improvements in HE and indicated trends toward improvements in ACLF grade, CLIF-C OF score, and need for vasopressors as documented in Table S2.

**Table 5:** Changes in Liver Function Parameters, ACLF Severity Scores and Organ Support Requirements Before and After the Final TPE Session in the TPE Group.

Parameter	Pre-TPE Median (IQR)	Post-TPE (24–48 h) Median (IQR)	*P*-Value
ACLF grade	2.5 (2–3)	1 (1–3)	0.0017
CLIF-C-ACLF score	55.5 (52.25–60.75)	47 (35.5–64.5)	0.039
HE (West Haven)	1 (0–2)	0 (0–1.25)	0.038
PT (%)	39% (24–46.5%)	39.5% (32–59.25%)	0.018
CLIF-C-OF score	12 (10–13)	10 (9–13.25)	0.084
Serum bilirubin (μmol/l)	328.5 μmol/l (106–485.55)	174.45 μmol/l (120.8–299.07)	0.064
Vasopressor need	45%	22.2%	0.098
Mechanical ventilation	35%	16.7%	0.083
Renal replacement therapy	60%	55%	1.0

ACLF, acute on chronic liver failure; CLIF-C, chronic liver failure consortium; HE, hepatic encephalopathy; hrs, Hours; OF, organ failure; PT, prothrombin time.

## Discussion

Systemic inflammation is the hallmark and driver of ACLF, resulting in functional multiorgan failures and a high short-term mortality rate. Besides proinflammatory overspill, patients suffer from cirrhosis-associated immune dysfunction and immune paralysis.^31^ Very few high-quality randomized therapy studies have explored the effects of extracorporal liver-directed support systems and therapies.^32^, ^33^, ^34^, ^35^, ^36^, ^37^ TPE addresses the aforementioned pathophysiological key concepts of hyperinflammation and immune paralysis in ACLF by diminishing proinflammatory cytokines, PAMPs and DAMPs, and replacing dysfunctional plasma components. It is, however, unselective, and the extent of filtration of potentially beneficial and protective plasma components is uncertain. Fernandez *et al.* demonstrated significant improvements of organ dysfunction in a small pilot-study in ACLF patients treated with TPE.^38^ The results of the large controlled randomized follow-up APACHE trial (NCT03702920) are pending. TPE has shown survival benefits in several ACLF-cohorts, mostly retrospective in nature.^19^, ^20^, ^21^, ^22^, ^23^, ^24^ However, it is important to note that the majority of these studies used the APASL or Chinese criteria to define ACLF and were performed in Asian populations. In the current study we applied the EF-CLIF criteria, which have been shown to have a stronger correlation with patient prognosis and outcomes in a European cohort.

In our study, patients with ACLF unresponsive to SMT after up to 7 days who subsequently received TPE showed a significant improvement in short-term transplant-free survival at 28 days, regardless of ACLF grade, compared to a well-matched cohort treated with SMT alone. Key parameters defining organ dysfunction in ACLF, such as coagulopathy and hepatic encephalopathy, demonstrated significant improvements, with the improvement in HE persisting 5–7 days after the final TPE session. There were also positive trends noted in reducing bilirubin levels, the need for vasopressors, and mechanical ventilation. TPE was overall well tolerated, with no serious adverse events reported.

Another notable observation was the wide interquartile range in the timing of TPE initiation after ACLF onset, spanning 7–17 days. While improvements in ACLF grade and CLIF-C scores may partially reflect the substitution of coagulation factors via TPE replacement fluid, this explanation is limited by the fact that reassessments were conducted at least 24 h after the last TPE session, exceeding the half-life of most coagulation factors. Furthermore, the observed short-term survival benefit cannot be attributed solely to coagulation factor replacement.

Waiting-list mortality for ACLF patients surpasses that of those listed for acute liver failure.^39^ Given the limited availability of organs, which frequently delays liver transplantation, there is a pressing need for therapies that stabilize organ function in ACLF patients, improve outcomes, and increase the feasibility of liver transplantation. Our observation that TPE improved ACLF grades in some patients suggests that it may help stabilize individuals initially considered too sick for transplantation, potentially making them eligible for this life-saving procedure.

It is important to emphasize, however, that the clinical indication for TPE in our study was neither primarily as a bridge to transplantation, nor was transplantation a designated endpoint of this investigation. Furthermore, the number of liver transplants in the TPE group was lower than that in the SMT group, indicating that TPE did not result in an improvement in transplant eligibility within our cohort. The decision to initiate TPE was made by the treating medical team on a case-by-case basis, which was not standardized and could introduce potential selection bias. Additionally, the retrospective nature of the study contributed to variability in the number of TPE sessions and their timing, potentially affecting the consistency of results. PSM was employed to address confounding factors. While PSM effectively eliminated statistically significant differences between both groups, it cannot be dismissed that unidentified factors may have obscured the true effect of TPE.

The limitations of our study are considerable and include its retrospective design, small sample size, and single-center setting. These factors introduce biases and significantly affect the generalizability of the findings. Furthermore, the small sample size and retrospective nature contribute to a strong selection bias, necessitating cautious interpretation of all results. Despite these constraints, significant findings at 28 days in both univariate and multivariate Cox regression analyses, along with the Kaplan–Meier survival function, indicate a potential short-term survival benefit for selected ACLF patients. However, the results at 90 days were discordant regarding the impact of TPE on survival and mortality, likely due to differing testing modalities.

While our study is limited by its methodology and the size of the study population, it mirrors real-world clinical practice and provides valuable insights into the routine use of TPE. It suggests that TPE may benefit selected subgroups of ACLF patients who are not responsive to standard medical care. This preliminary finding warrants further investigation through prospective, randomized trials. Our findings contribute to the limited existing data on this topic, aiming to encourage further research into the potential role of TPE in managing ACLF.

## Funding

No financial support of funding was provided for this work.

## Credit authorship contribution statement

Conceptualization JS, AH and TB. Methodology and formal analysis JS and JF. JS, TB, AH, RV, SP, LW and RB were involved in patient's care and sample collection. JS and JF prepared the figures. JS, RB and TB wrote the manuscript. All authors revised and approved the final manuscript.

## Declaration of competing interest

The authors declare that they have no known competing financial interests or personal relationships that could have appeared to influence the work reported in this paper.
