# Supplementary material for: Exploring the Impact of Therapeutic Plasma Exchange on Organ Function in Patients With ACLF: A Retrospective, Single-center Propensity Score-matched Cohort Study
Source: J Clin Exp Hepatol. 2025 Mar 24;15(5):102550. doi: 10.1016/j.jceh.2025.102550 (PMC12490706; doi:10.1016/j.jceh.2025.102550)
Supplement: Multimedia component 1 [file mmc1.docx]

| Patient | Sex  (M=male)  (F= Female) | Age  (years) | Weight  (kg) | Height  (cm) | TPE-Sessions (n) | Rhythm | Replacement volume per session (ml)** | Replacement  Fluids |
| --- | --- | --- | --- | --- | --- | --- | --- | --- |
| 1 | M | 46 | 92 | 187 | 3 | Daily | 4900/4900/4900 | FFP |
| 2 | M | 65 | 105 | 175 | 3 | Daily | 4000/4000/4000 | FFP |
| 3 | M | 53 | 102 | 189 | 2 | Daily | 4400/4400 | FFP |
| 4 | M | 49 | 116 | 185 | 2 | Daily | 6000/6000 | FFP |
| 5 | M | 65 | 75 | 176 | 10 | 7x Daily  3x every other Day | 3500/4000/4000/4000/4000/4000/4000/  4000/4000/4000 | FFP |
| 6 | M | 65 | 80 | 170 | 2 | Daily | 4400/4200 | FFP |
| 7 | M | 56 | 103 | 185 | 3 | Daily | 3900/4200/4200 | FFP |
| 8 | M | 62 | 70 | 170 | 3 | Daily | 3400/3600/3500 | FFP |
| 9 | M | 62 | 89 | 178 | 6 | every other Day | 3500/4100/4400/4500/4400/4200 | FFP |
| 10 | F | 41 | 61 | 171 | 6 | 4x Daily,  2x every other Day | 3800/3400/3400/3400/  3400/3200 | FFP |
| 11 | F | 60 | 63 | 162 | 5 | Daily | 2900/2800/300/2800/2900 | FFP |
| 12 | M | 29 | 102 | 175 | 5 | Daily | 5700/6200/6000/6000/6000 | FFP |
| 13 | F | 71 | 57 | 158 | 2 | Every other Day | 3000/2900 | FFP + Albumin 5% |
| 14 | F | 41 | 75 | 168 | 7 | Every other Day | 3500/3500/3500/3500/3500/3500/3300 | FFP + Albumin 5% |
| 15 | M | 35 | 71 | 191 | 3 | Every other Day | 4800/4500/4500 | FFP + Albumin 5% |
| 16 | M | 68 | 103 | 178 | 3 | Every other Day | 4900/4800/4800/4600/4700/4800 | FFP + Albumin 5% |
| 17 | F | 48 | 92 | 165 | 2 | Daily | 3600/3600 | FFP |
| 18 | F | 34 | 97 | 170 | 3 | Daily | 4000/4000/4000 | FFP |
| 19 | F | 29 | 110 | 175 | 5 | Daily | 4300/4300/4300/4300/4300 | FFP + Albumin 5% |
| 20 | F | 34 | 83 | 176 | 5 | Daily | 3900/3800/3900/3700/3900 | FFP |

* rounded values

**Table S1:** The table displays the detailed replaced plasmavolume and substitution volume for each treated patient in the TPE group. The replaced volume was rounded to hundreds. FFP, fresh frozen plasma.

| **Parameter** | **Pre-TPE**  **Median (IQR)** | **Post-TPE (Day 5-7)**  **Median (IQR)** | **p-Value** |
| --- | --- | --- | --- |
| ACLF Grade | 2.5 (2-3) | 2 (1-2.75) | 0.054 |
| CLIF-C-ACLF Score | 55.5 (52.25-60.75) | 51 (42.5-58) | 0.184 |
| HE (West Haven) | 1 (0-2) | 0 (0-1) | 0.026 |
| PT (%) | 39% (24-50%) | 37% (24.75-67%) | 0.191 |
| CLIF-C-OF Score | 12 (10-13) | 10 (8-11.75) | 0.088 |
| Serum Bilirubin (µmol/l) | 328.5 µmol/l (106-485.55) | 218 µmol/l (93.52-336.57) | 0.191 |
| Vasopressor Need | 45% | 22.2% | 0.098 |
| Mechanical Ventilation | 35% | 16.7% | 0.157 |
| Renal replacement therapy | 60% | 55% | 0.317 |

**Table S2:** Changes in liver function parameters, ACLF severity scores and organ support requirements before and 5-7 days after the final TPE session in the TPE group. (ACLF, acute on chronic liver failure; CLIF-C, chronic liver failure consortium; HE, hepatic encephalopathy; OF, organ failure; PT, prothrombin time)
