# Supplementary figures and images for: Exploring the Impact of Therapeutic Plasma Exchange on Organ Function in Patients With ACLF: A Retrospective, Single-center Propensity Score-matched Cohort Study
Source: J Clin Exp Hepatol. 2025 Mar 24;15(5):102550. doi: 10.1016/j.jceh.2025.102550 (PMC12490706; doi:10.1016/j.jceh.2025.102550)

## SMT + TPE

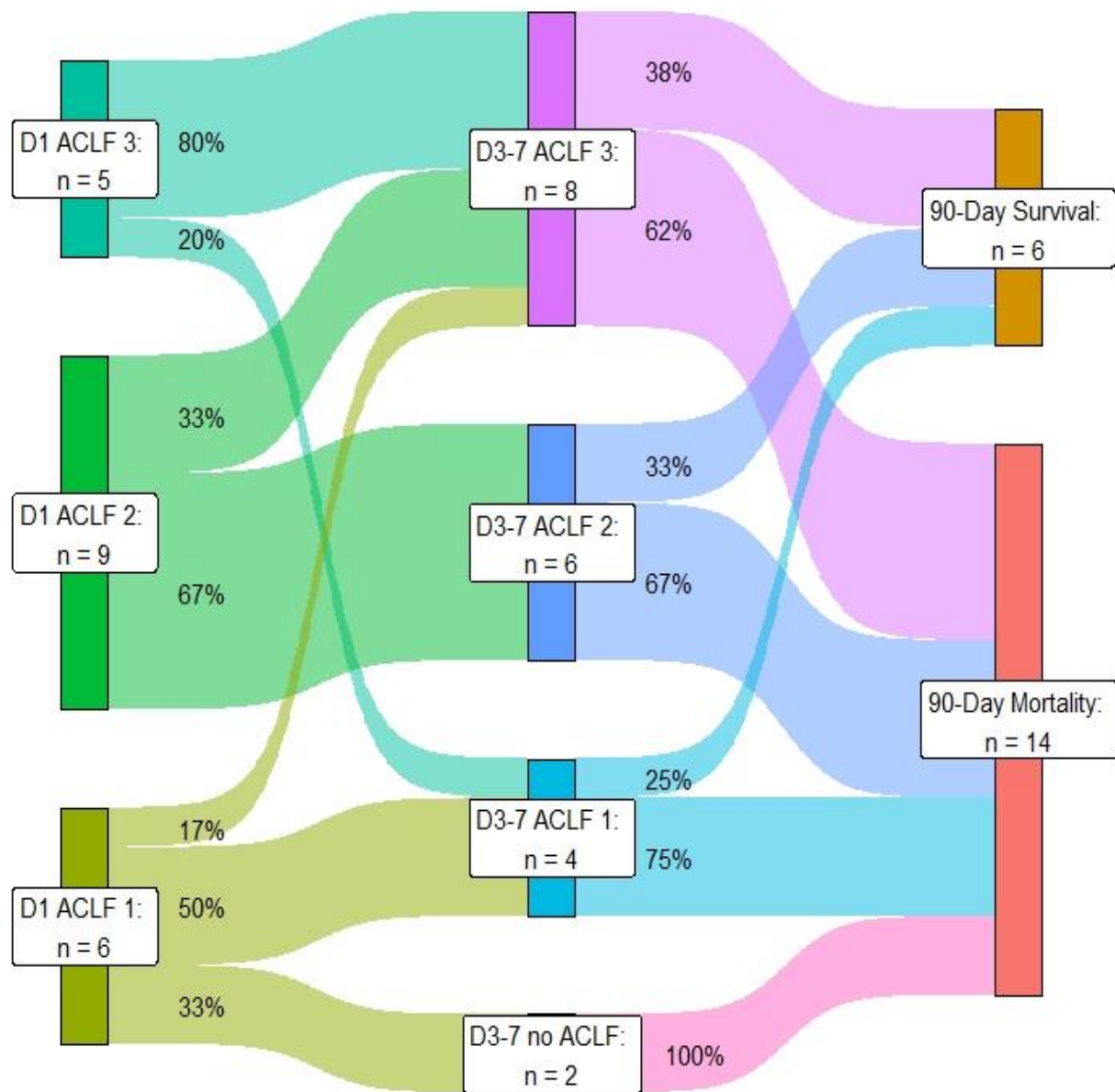

## SMT

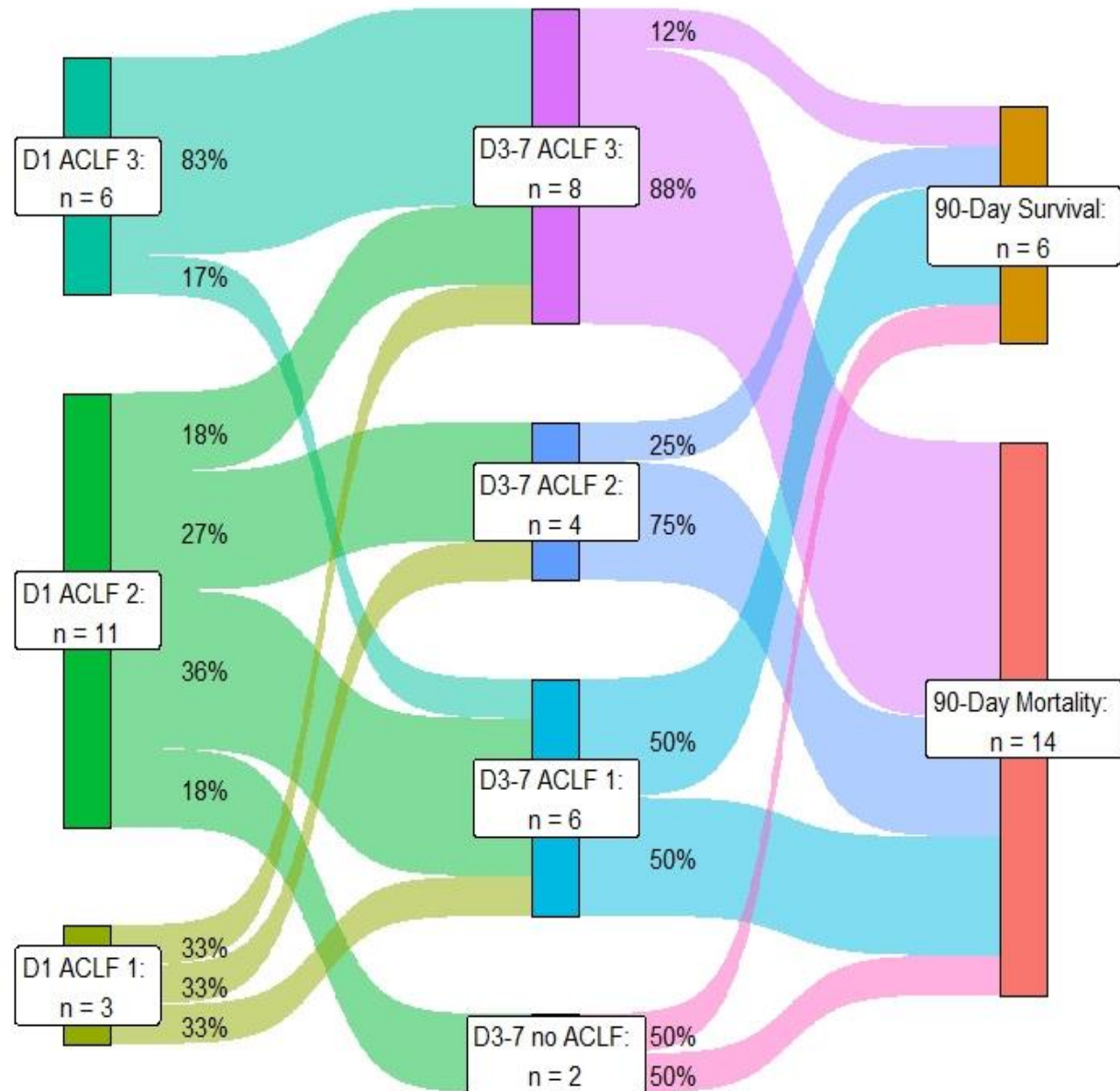

Supplement: Multimedia component 2 [file mmc2.pdf]

### 90-Day Tx-free Survival

+ SMT + SMT+TPE

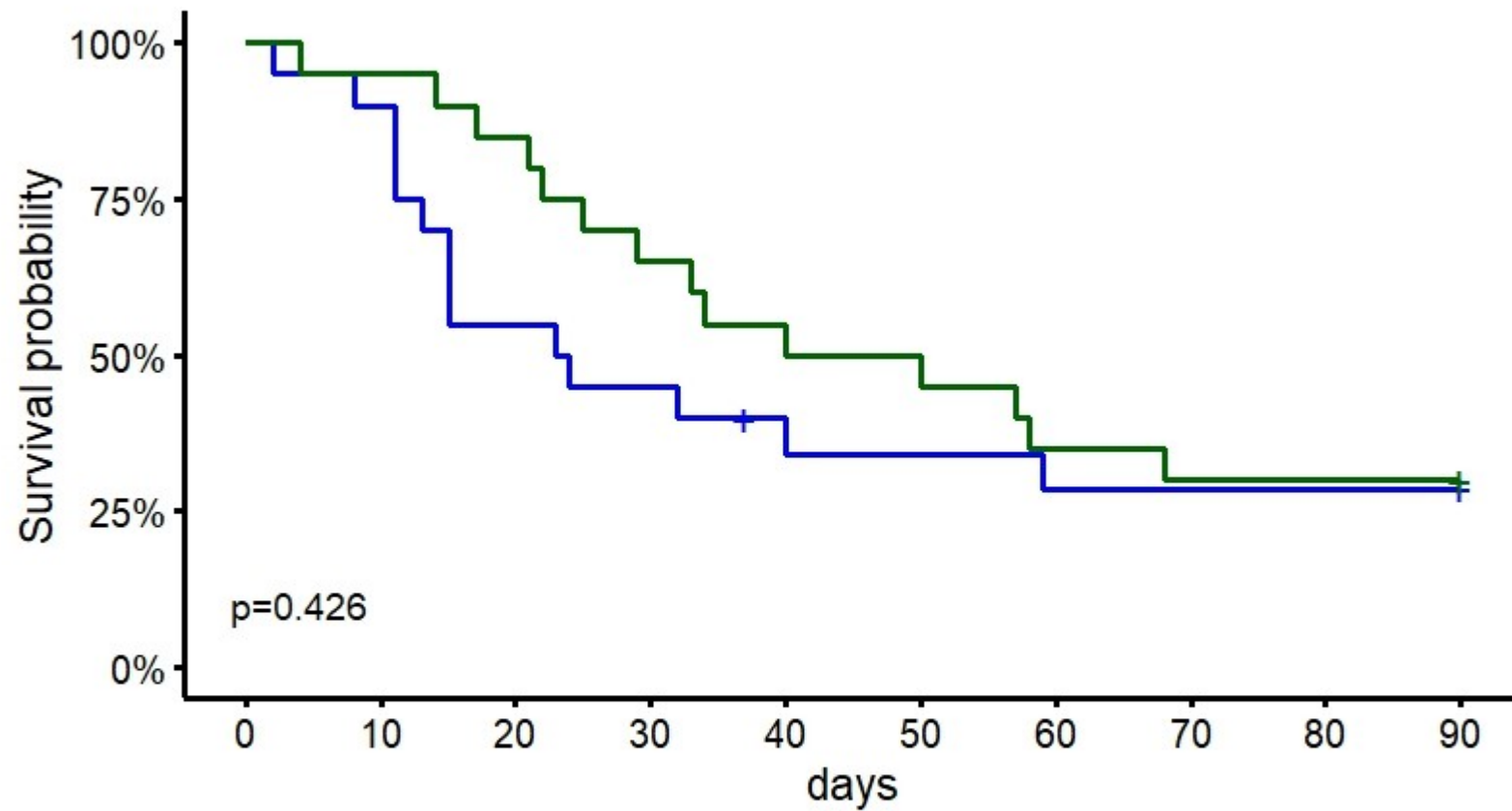

### Patients at risk

|         |    |    |    |    |    |    |   |   |   |   |
|---------|----|----|----|----|----|----|---|---|---|---|
| SMT     | 20 | 18 | 11 | 9  | 7  | 6  | 5 | 5 | 5 | 5 |
| SMT+TPE | 20 | 19 | 17 | 13 | 11 | 10 | 7 | 6 | 6 | 6 |

Supplement: Multimedia component 3 [file mmc3.pdf]
