# Supplementary material for: Exploring the Impact of Therapeutic Plasma Exchange on Organ Function in Patients With ACLF: A Retrospective, Single-center Propensity Score-matched Cohort Study
Source: J Clin Exp Hepatol. 2025 Mar 24;15(5):102550. doi: 10.1016/j.jceh.2025.102550 (PMC12490706; doi:10.1016/j.jceh.2025.102550)
Supplement: Multimedia component 4 [file mmc4.pdf]

90-Day Tx-free Survival in patients without improvement of ACLF-Grade after 3-7 Days

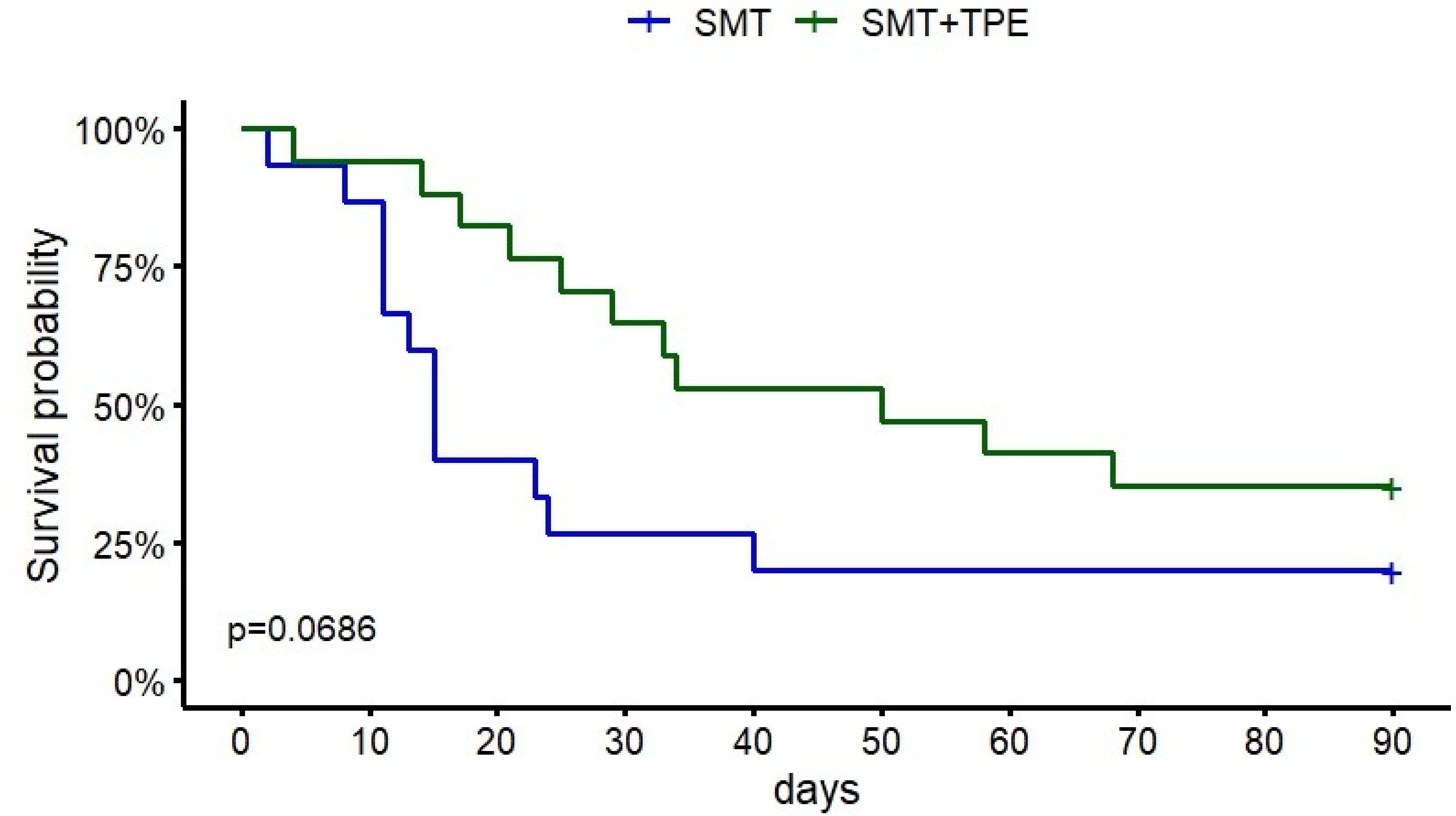

Patients at risk

	0	10	20	30	40	50	60	70	80	90
SMT	15	13	6	4	4	3	3	3	3	3
SMT+TPE	17	16	14	11	9	9	7	6	6	6
